# Biosynthesis of bimetallic and core–shell nanoparticles: their biomedical applications – a review

**DOI:** 10.1049/iet-nbt.2017.0308

**Published:** 2018-06-21

**Authors:** Mehrdad Khatami, Hajar Q. Alijani, Iraj Sharifi

**Affiliations:** ^1^ NanoBioElectrochemistry Research Center, Bam University of Medical Sciences Bam Iran; ^2^ Leishmaniasis Research Center Kerman University of Medical Sciences Kerman Iran; ^3^ Research Center of Tropical and Infectious Diseases, Kerman University of Medical Sciences Kerman Iran

**Keywords:** nanofabrication, nanoparticles, biosensors, microorganisms, molecular biophysics, nanobiotechnology, biosynthesised bimetallic –shell, environmental friendliness, green synthesis, eco‐friendly efficient synthesis process, core–shell nanostructures, bimetallic –shell nanostructures, physiochemical methods, nanostructures synthesis, green physiochemical, NPs science, hybrid NPs, biomedical applications, core–shell nanoparticles, bimetallic –shell nanoparticles, biosynthesis

## Abstract

Recently, researchers succeeded in designing and manufacturing a new class of nanoparticles (NPs) called hybrid NPs. Among hybrid NPs, bimetallic and core–shell NPs were a revolutionary step in NPs science. A large number of green physiochemical and methods for nanostructures synthesis have been published. Eventually, physiochemical methods are either expensive or require the use of chemical compounds for the synthesis of bimetallic and core–shell nanostructures. The main challenges that scientists are facing are making the process cheaper, facile and eco‐friendly efficient synthesis process. Green synthesis (biosynthesis) refers to the use of bio‐resources (such as bacteria, fungi, plants or their derivatives) for the synthesis of nanostructures. The popularity of the green synthesis of nanostructures is due to their environmental friendliness and no usage of toxic materials, environmental friendliness for the synthesis or stability of nanostructure. Bimetallic and core–shell NPs have many biomedical applications such as removing heavy metals, parasitology, molecular and microbial sensor, gene carrier, single bacterial detection, oligonucleotide detection and so on. The purpose of this study is to discuss briefly the biosynthesised bimetallic and core–shell NPs, their biomedical applications.

## 1 Introduction

**Fig. 1:**
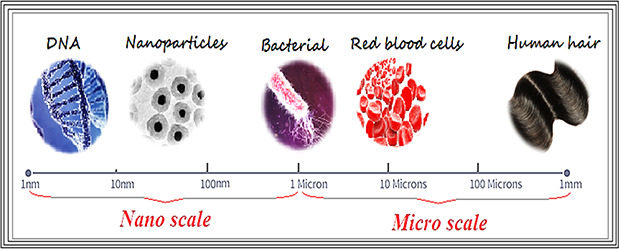
Nano to micro scales

Nanoparticles (NPs) are defined as a particle with size range in 1–100 nm, at least for one dimension [1]. Materials that are sized at NPs are about 1000 times smaller than microsized (micronsized) particles (Fig. 1). NPs have shown novel biological and physicochemical properties [2, 3, 4, 5, 6, 7, 8].

At first, the main focus of scientists was the synthesis and application of NPs that contained single structures, called simple NPs such as silver, gold and selenium, due to their unique features and utilisation [9, 10, 11, 12, 13, 14, 15, 16, 17].

**Fig. 2:**
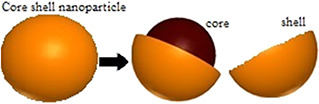
Structure of core–shell NPs

The improvement of knowledge has helped scientists to design a new class of NPs known as hybrid NPs, which can be defined as well‐organised nanomaterials consisting of two, three, or more types of single nanocomponents [18]. Core–shell NPs are a kind of hybrid NPs that are also written as core/shell, core–shell, and core@shell NPs. Core–shell NPs are composed of two or more nanomaterials, which includes a wide range of organic and inorganic nanomaterials (metals or polymers), while one of them acts as a core and the other nanomaterial is located around the central core called shell (Fig. 2). Bimetallic NPs composed of two different metal elements [19].

The knowledge of hybrid NPs synthesis stands as a revolutionary step in the nanoscience. The ability to manipulate NP's structure has helped us in producing a large number of hybrid NPs [20, 21, 22, 23, 24, 25]. The core–shell NPs with the ability to be utilised in a wide range of materials as core or shell can represent its satisfying unique features and custom functions. Depending on the purpose of the study, core or shell materials can be selected [26]. The properties of core–shell NPs can be altered by inducing changes in the ingredients that constitute the core or shell layer. Features and distinctive properties including physicochemical, biological, optical, etc. can be observed when different nanomaterials are combined such as core shell NPs. These hybrid NPs are employed in designing applicable programs in different fields such as medicine, engineering and so on [27, 28, 29, 30, 31].

**Fig. 3:**
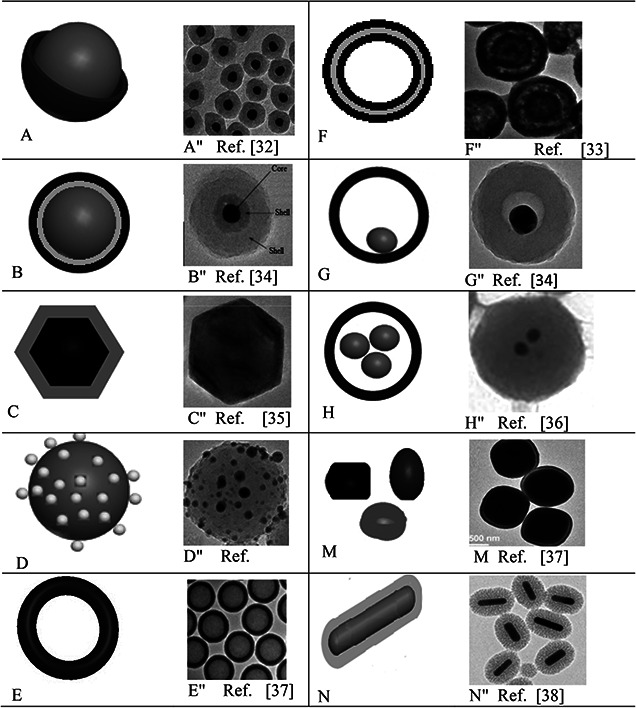
Schematic diagram (A–H) and TEM (A″–H″) pictures of different structures of core–shell NPs A, A″: core–shell NPs B, B″: core double‐shell particles or core multi‐shell NPs C, C″: polyhedral core/shell NPs D, D″: core porous‐shell NPs E, E″: hollow‐core shell NPs or single‐shell NPs F, F″: hollow‐core double‐shell NPs G, G″: moveable‐core–shell NPs H, H″: multi‐core–shell NPs M, M″: irregular shape core–shell NPs N, N″: rod core–shell NPs

Core–shell NPs are classified into the following types (Fig. 3, [32, 33, 34, 35, 36, 37, 38]) based on the structure [26].

Each structure contains its own unique and specific properties.

## 2 Different synthesis methods of hybrid (core–shell) NPs

A large number of bottom‐up and top‐bottom approaches have been reported for the synthesis of various nanomaterials.

In the bottom‐up approach, self‐assemble type of atoms led to the formations of nanosized particles; but in top‐bottom approach, bulk materials are broken down to nanosized particles (has not been yet put under investigation for core–shell NPs). Although the top‐bottom approach is an applicable technique, yet the bottom‐up methods are preferred.

**Fig. 4:**
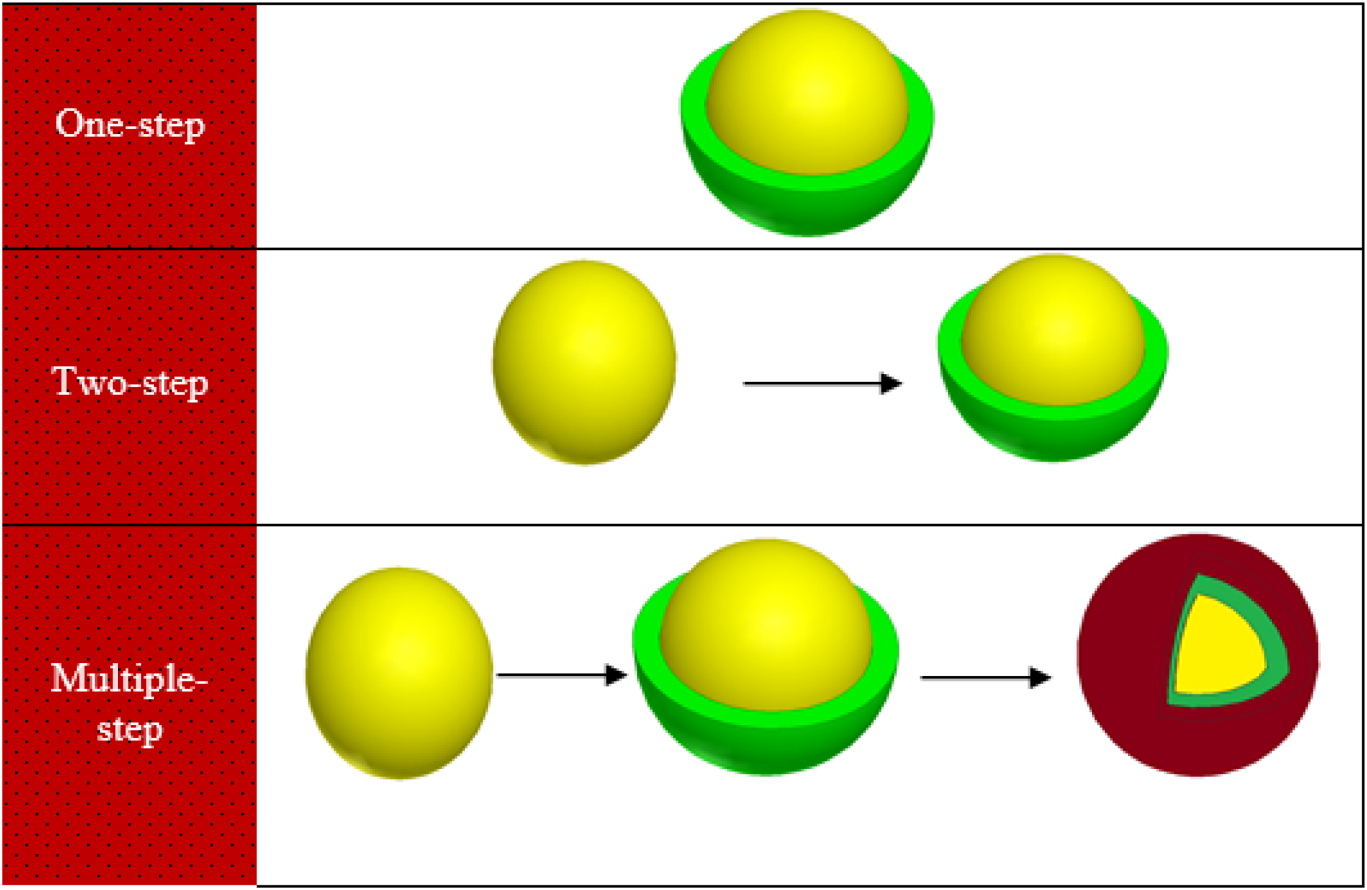
Schematic of one‐step, two‐step and multiple‐step synthesis approaches of core–shell NPs

Regarding the synthesis of core–shell NPs, three step approaches have been reported:

i. *One‐step:* The core and shell are formed together.
ii. *Two‐step* : The core is synthesised first, then the shell layer is formed around the synthesised core surface.
iii. *Multiple‐step:* The core is synthesised first, then the first shell layer is formed around the synthesised core surface and finally a second shell layer is set up on the first shell surface or have the core removed, in which hollow‐core–shell NPs can stand (Fig. 4).

**Fig. 5:**
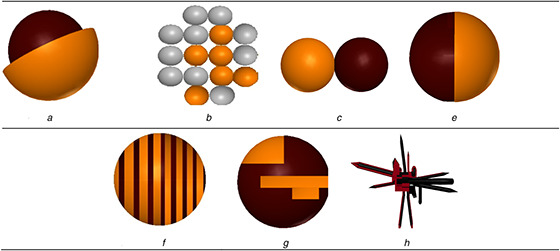
Schematic diagram (a–f) images of different structures of bimetallic NPs ***(a)*** Core–shell NPs (all structures of core–shell NPs such as bimetallic moveable‐core–shell NPs and so on), ***(b)*** Random mixed structure bimetallic NPs, ***(c)*** Dumbbell structure, ***(e)*** Structure with two interfaces, ***(f)*** Regular mosaics, ***(g)*** Irregular mosaics, ***(h)*** Random mixed dendritic structure (sometimes named cluster, star or flower shape structure)

Based on the structure, bimetallic NPs are classified into the following types (Fig. 5) (it should be mentioned that bimetallic NPs are not necessarily spherical. The following bimetallic pictures are samples for help to better understanding of bimetallic NPs structure):

Based on the essence, the hybrid (bimetallic or core shell) NPs synthesis methods are classified to: physical, chemical, green synthesis or combination of above two or three methods [39]. Although each of these techniques has their own advantages and disadvantages, yet the green synthesis methods have proven to be cost‐effective, environmentally benign and easier than physiochemical methods. Nature has provided millions of bio‐resources (fungi, bacteria, plants or their derivatives) that seem to be suitable for the synthesis of a wide range of nanostructures [40]. The advantage of nanostructures synthesised by green approach is that these particular bio‐resources contain a large variety of biomolecules that cover the surface of synthesised NPs, thus forms the capping layers around it, which further provide stability and biocompatibility to green NPs. Although one of the challenges that are faced regarding most of the physically and chemically synthesised NPs is aggregation, yet it seems the biomolecules that cover the surface of green synthesised NPs are capable of avoiding aggregation due to existing capping layer [41, 42]. In comparison with the plant extract, the production of bimetallic and core–shell NPs through the utilisation of bacteria and fungi are very limited. Up to now, less than ten studies have been performed on bacterial or fungi biosynthesis of hybrid core–shell NPs. Despite the fact that it is possible to use bacteria or fungi strains in the process of synthesising NPs, yet researchers are not interested due to its difficulties and dependency on costly cell cultures.

Several published reports have demonstrated the biosynthesis of bimetallic and core–shell NPs such as magnetic nickel/iron‐oxide, gold/silver, Fe_3_ O_4_ –Ag and Au–Ag NPs. Plants or their derivatives are suitable for simple, fast, and stable synthesis of bimetallic and core–shell NPs with different shapes, structures, and sizes in large scales.

**Fig. 6:**
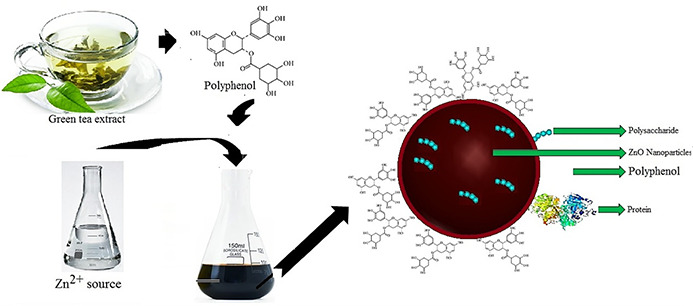
Schematic for the synthesis of NPs using green tea that led to the synthesis of polyphenol surface coating NPs

If the desired surface structure of green synthesised NPs is obtained, they can be forced to bind to a variety of polymers, antibodies, aptamers and so on. In the green synthesis of nanostructures, the extract has a significant influence on the coated functional groups that exist on the surface of resulting synthesised nanostructures. Depending on the bio‐resource applied in the green synthesis process of nanostructures, the surfaces can be modified by different functional groups. Therefore, choosing the right bio‐resource is critical for achieving the suitable surface that is modified by the obtained synthesised nanostructures. For example, the use of green tea leaf extract, due to its high polyphenol content can lead to a polyphenol surface coating on the resulting synthesised nanostructures (Fig. 6).

Therefore, it can be noted that depending on the purpose, the green synthesis of NPs is possible and practicable. Selecting the proper bio‐resource for the synthesis procedure in addition to the reducing ions for the nanostructures can modify the surface coating of NPs. The biosynthesised core–shell NPs have had a better usage in clinical trials. Chemically synthesised NPs may end up coated with toxic chemical materials that are applied in the synthesis process, limiting their applications in medical studies by considering how these impurities can be involved in the experiments. However, biosynthesising NPs have brought a solution for these restrictions. For example, some NPs are rapidly oxidised such as copper and iron. One way to prevent oxidisation is to cover their surface by the shell layer.

## 3 Characterisation of bimetallic and core–shell NPs

Identifying and studying the physicochemical properties, structure details, purities, and dopants of biosynthesised NPs is very important as the structure, size, shape of NPs can significantly affect their performance and properties. In fact, determining the physicochemical characteristics of NPs helps to effectively understand the relationship between these characteristics and their performance. From previous studies, the antimicrobial efficiency of silver NPs with smaller sizes seem to be more than that of the larger sizes. Describing the behaviour and structure of green synthesised NPs is quite difficult due to the existence of various macromolecules in their extract which participate in their own structure.‏

In the in following sections, common physicochemical characterisation techniques of NPs are described briefly.

### 3.1 Spectroscopic analysis

UV–vis spectroscopy analysis is very common for primary detection of different kinds of NPs with the ability to absorb electromagnetic radiation in the UV–vis spectral region; e.g. the UV–vis absorbance of gold NPs is around 490–600 nm range.

### 3.2 Microscopic analysis

Microscopic analyses, such as transmission electron microscopy (TEM) and high‐resolution TEM (HRTEM), atomic‐force microscopy (AFM), scanning electron microscopy (SEM), field emission SEM (FESEM) and analysis have been used to examine the size, morphology, and distribution of nanomaterials. AFM and SEM images are not applicable for studying the structure of core–shell NPs since they characterise the surfaces. Recognising the core in SEM images is too difficult while TEM images are very convenient for studying the structure of core–shell NPs considering its ability in measuring the thickness and spacing between core and shell/shells. Energy dispersive spectrometry (EDS) is an accessory of electron microscopy instruments (TEM and SEM). EDS is a powerful method for determining the chemical nature of the core and shell, which has displayed the distribution of elements in the studied samples.

### 3.3 X‐ray diffraction (XRD)

Scattering analysis and XRD analysis have been utilised to examine the crystal structure and phase purity of the synthesised NPs (crystal structure is a description of the ordered arrangement of atoms).

### 3.4 Fourier transform infrared (FTIR) spectroscopy

FTIR can be used to identify the surface modification of NPs, confirm the load‐drug and overlay functional, identify the type of functional groups and biomolecules that are responsible for capping and efficient stabilisation of NPs, ensure the existence of shells in core–shell NPs, verify the band between the two layers of the shell in core double shell NPs, and qualitative and quantitative identification of the molecular structure of organic compounds in the NPs structure and especially in structure of core–shell NPs or hollow‐core/porous‐shell materials.

### 3.5 Thermal gravimetric analysis (TGA)

TGA is a thermal analysis by which the mass of an NPs is measured over time as the temperature changes (usually between 25 and 800°C). Also, the properties of the oxidation resistance of the core–shell NPs can be tested by the TGA. For example, Ammar *et al.* illustrated that the weight increment of the coated particles caused by FeNi oxidation decreased from 20 to 4%, which is relative to that of the uncoated FeNi particles using TGA.

This analysis can be utilised to determine the structure of core–shell NPs, hydration, effective absorption of drugs in mesopore NPs, and measuring the magnetic performance of hybrid NPs. TGA measurements can be performed under different atmospheres such as air, hydrogen, ozone, and argon. We can ascertain the amount of organic molecule residues in the structure of NPs through the TGA analysis in ozone. Significant weight loss in the mass of green synthesised NPs at high temperatures is due to the degradation of biomolecules in the structure of NPs, considering how the biomolecules in the extract play the roles of both capping and reducing agents.

### 3.6 Vibrating sample magnetometer (VSM)

VSM can be used to study the magnetic properties of NPs. Maintaining magnetic properties, achieving higher magnetic properties, determination of the magnetic performance regarding the core–shell NPs when compared to the single structured NPs, have made it important and practicable in biology, medicine and industry applications. The magnetic Fe_3_ O_4_ : TiO_2_ core–shell NPs can be applied to tumour therapy. VSM has shown the magnetic properties of iron–iron derivatives NPs with higher magnetism than iron NPs (single structure).

### 3.7 X‐ray photoelectron spectroscopy (XPS)

It is very vital to identify the core–shell NPs oxidation status in catalytic systems and gas detection sensors in order to understand their chemical and physical behaviours. The surface oxidation of these NPs has been investigated via XPS analysis, which is a technique for analysing the surface elements with a nanometre sampling depth. It can also determine the atomic ratio in NPs with heterogeneous structure and provide the chemical information of specified elements such as distinguishing between sulphate and sulphide forms of sulphur that necessary for comprehending the morphology of NPs core–shell.

### 3.8 Brunauer–Emmett–Teller (BET)

Precise measurement of surface area, volume, and pore distribution is important in characterising the polymers pharmaceutical materials and the coating of NPs. BET analysis was used to determine the structure of porous, as well as the shape and position of the cavities that are relative to each other within the NPs texture. The surface specific area, peculiarities of the surface, and volume of the pores of the hybrid NPs play a vital role in determining their functional activities such as the amount of drug loaded in NPs‐based targeted drug delivery systems, and controlling the release of the loaded drug, absorption, storage, catalytic and so on.

## 4 Biomedical applications of core–shell NPs

**Fig. 7:**
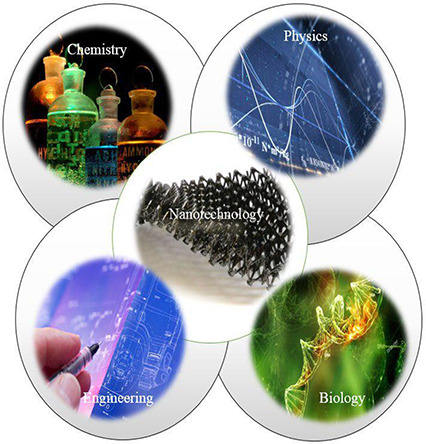
Schematic relationship between nanoscience and others sciences

Nowadays, nanoscience stands as one of the most attractive sciences in the world (Fig. 7). Nanoscience cannot be limited to a specific category. This particular area is an interdisciplinary field of science that can be employed for many applications [43, 44, 45, 46, 47, 48, 49, 50].

Compared to single NPs, bimetallic and core–shell NPs with improved properties have a special economic value due to the existing increase in their durability, performance, and breadth of applications in medicine, engineering and other industrial fields. In recent years, core–shell NPs have caught the attention of scientists due to the diversity of their structures, potential and multipurpose applications, unique structural features, simple production methods and easy control. These NPs contain several beneficial features such as the ability to function in a wide range of pH, temperatures, magnetic properties and so on [51, 52, 53, 54, 55].

**Fig. 8:**
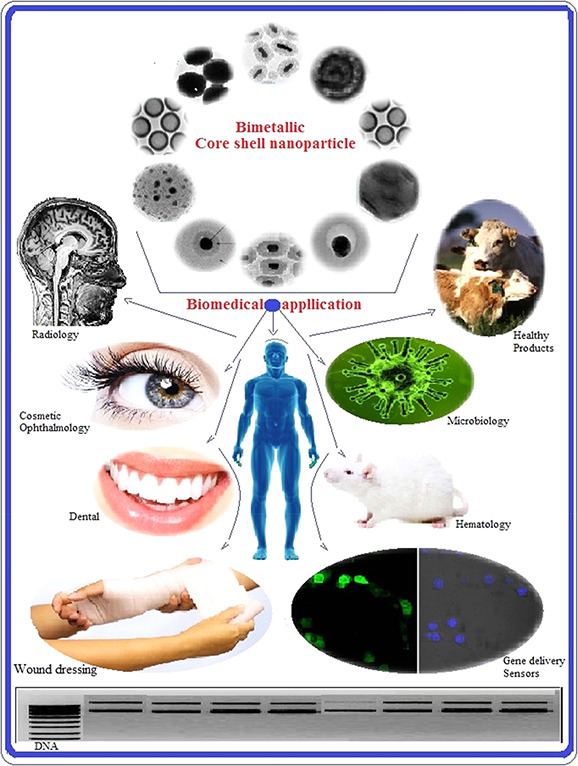
Applications of biosynthesised bimetallic and core–shell NPs in biomedical fields

Design and synthesis of hybrid NPs with desired structures can attract the attention of scientists toward biosynthesis hybrid NPs. The designs of suitable custom hybrid NPs and their utilisations are truly endless. Some products will be offered every day in this area while containing a very strong economic ripe. A hybrid has been known to be selective and sensitive when used as DNA, protein, secondary metabolic or enzyme markers for diagnosing pathogens cells or diseases. Medical applications of hybrid NPs help in the early diagnosis of pathogens or diseases (Fig. 8). Table 1 summarises the biomedical applications of different hybrid NPs (Table 1).

**Table 1:** Some biomedical applications of different hybrid NPs

No.	Hybrid nanostructure	Synthesis method	Application	Ref.
1	iron–copper bimetallic	chemical	degradation contaminants	[56]
2	Ag–Cu bimetallic	biosynthesis	cellular imaging	[57]
3	Au–Ag bimetallic	biosynthesis	degradation of harmful dye	[58]
4	NiZnO nanocomposite	chemical	removal of toxic textile dyes from wastewater	[59]
5	ZnO@polymer core–shell	biosynthesis	cell imaging	[60]
6	Ni/NiO core Shell	chemical	protein separation	[61]
7	Co@Au yolk/shell nanospheres	—	gene transport vehicles, cellular optical imaging	[62]
8	magnetic luminescent core shell	chemical	immunoassays	[63]
9	cobalt ferrite core–shell	chemical	peptide nucleic acid and DNA biosensor	[64]
10	palladium/platinum	biosynthesis	drug carrier and cancer treatment	[65]
11	Au–Ag core–shell	biosynthesis	antibiofilm and antileishmanial activity	[66]
12	Au–Ag bimetallic	biosynthesis	drags detection	[67]
13	silica core–shell	—	MRI contrast and cancer imaging	[68]
14	platinum–gold alloys	—	biofuel cells	[69]
15	CuZn bimetallic	chemical	fungicides	[70]
16	Au–Ag bimetallic	biosynthesis	biosensing, bioimaging and biomedicine	[71]
17	CuFe bimetallic	chemical	antibacterial	[72]
18	Au–Ag bimetallic	chemical	detection of disease biomarkers	[73]
19	copper and nickel bimetallic	chemical	enhanced bacterial inhibition	[74]
20	Au–Ag bimetallic	biosynthesis	antibacterial	[75]
21	Pt–Pd bimetallic	—	detection of xanthine	[76]
22	Cu Ni and Cu Ag bimetallic	biosynthesis	degradation water contaminants	[77]
23	copper–silver bimetallic	biosynthesis	antibacterial	[78]
24	zero‐valent iron/Cu	—	remove hexavalent chromium from groundwater	[79]
25	Cu@Pt core–shell	—	antimicrobial	[80]
26	Au–Pd core–shell	chemical	detection of epinephrine	[81]
27	Au–Ag bimetallic	—	antifungal activity	[82]
28	Pt–Au bimetallic	—	drug delivery	[83]
29	zinc oxide/silver bimetallic	—	antibacterial	[84]
30	gold‐coated iron oxide	—	photothermal therapy of cancer	[85]
31	Ag@Pd core–shell	biosynthesis	anti‐cancer/anti‐microbial	[86]
32	manganese ferrite core–shell	chemical	determination of tryptophan	[87]
33	Cu–Ni bimetallic	—	dentist	[88]
34	core–shell Fe_3_ O_4_ –Au	—	immunosensor	[89]
35	Fe_3_ O_4_ @SiO_2_ @Ag triple core–shell	—	sensor for adipokines detection	[90]
36	Fe_3_ O_4_ @Ag	—	biosensors	[91]

## 5 Conclusion

This review suggests the sustainable development in the green synthesis of all kinds of hybrids, especially core–shell NPs, which can lead to the expansion of green chemistry in near future.‏

The strong belief in the usage of biological resources (green synthesis) is caused by observing, chemical stability, solubility and biocompatibility of the synthesised NPs in water when compared to the conventional physicochemical methods. Green synthesis methods can improve human and environment health by helping in developing green chemistry and economic growth.
